# Intensity- and Duration-Adaptive Functional Electrical Stimulation Using Fuzzy Logic Control and a Linear Model for Dropfoot Correction

**DOI:** 10.3389/fneur.2018.00165

**Published:** 2018-03-19

**Authors:** Guangtao Chen, Zhihang Shen, Yu Zhuang, Xiaoyun Wang, Rong Song

**Affiliations:** ^1^Key Laboratory of Sensing Technology and Biomedical Instrument of Guangdong Province, School of Engineering, Sun Yat-Sen University, Guangzhou, China; ^2^The Guangdong Work Injury Rehabilitation Center, Guangzhou, China

**Keywords:** fuzzy logic control, linear model, dropfoot, functional electrical stimulation, treadmill

## Abstract

Functional electrical stimulation (FES) is important in gait rehabilitation for patients with dropfoot. Since there are time-varying velocities during FES-assisted walking, it is difficult to achieve a good movement performance during walking. To account for the time-varying walking velocities, seven poststroke subjects were recruited and fuzzy logic control and a linear model were applied in FES-assisted walking to enable intensity- and duration-adaptive stimulation (IDAS) for poststroke subjects with dropfoot. In this study, the performance of IDAS was evaluated using kinematic data, and was compared with the performance under no stimulation (NS), FES-assisted walking triggered by heel-off stimulation (HOS), and speed-adaptive stimulation. A larger maximum ankle dorsiflexion angle in the IDAS condition than those in other conditions was observed. The ankle plantar flexion angle in the IDAS condition was similar to that of normal walking. Improvement in the maximum ankle dorsiflexion and plantar flexion angles in the IDAS condition could be attributed to having the appropriate stimulation intensity and duration. In summary, the intensity- and duration-adaptive controller can attain better movement performance and may have great potential in future clinical applications.

## Introduction

Stroke is a leading cause of disability in the lower limb, such as dropfoot (1). A typical cause of dropfoot is muscle weakness, which results in a limited ability to lift the foot voluntarily and an increased risk of falls (2–4). Great effort is made toward the recovery of walking ability for poststroke patients with dropfoot, such as ankle–foot orthoses (5), physical therapy (6), and rehabilitation robot (7).

Functional electrical stimulation (FES) is a representative intervention to correct dropfoot and to generate foot lift during walking (8, 9). The electrical pulses were implemented *via* a pair of electrodes to activate the tibialis anterior (TA) muscle and to increase the ankle dorsiflexion angle. The footswitch or manual switch was used to time the FES-assisted hemiplegic walking in previous studies, while they were only based on open-loop architectures. The output parameters of the FES required repeated manual re-setting and could not achieve an adaptive adjustment during walking (10, 11). Some researchers have found that the maximum ankle dorsiflexion angle by using FES with a certain stimulation intensity had individual differences due to the varying muscle tone and residual voluntary muscle activity and varied during gait cycles (12, 13). If the stimulation intensity was set to a constant value during the whole gait cycle, the result could be that the muscle fatigues rapidly (14). Another important problem was that the FES using fixed stimulation duration from the heel-off event to the heel-strike event would affect the ankle plantar flexion angle (15, 16).

Closed-loop control was an effective way to adjust the stimulation parameters automatically, and several control techniques have been proposed (17, 18). Negård et al. applied a PI controller to regulate the stimulation intensity and obtain the optimal ankle dorsiflexion angle during the swing phase (19). A similar controller was also used in Benedict et al.’s study, and the controller was tested in simulation experiments (20). Cho et al. used a brain–computer interface to detect a patient’s motion imagery in real time and used this information to control the output of the FES (21). Laursen et al. used the electromechanical gait trainer Lokomat combined with FES to correct the foot drop problems for patients, and there were significant improvements in the maximum ankle dorsiflexion angles compared to the pre-training evaluations (22). There were also several studies that used trajectory tracking control to regulate the output and regulate the pulse width and pulse amplitude of the stimulation (23). The module was based on an adaptive fuzzy terminal sliding mode control and fuzzy logic control (FLC) to determine the stimulation output and force the ankle joint to track the reference trajectories. In their study, FES applied to TA was triggered before the heel-off event. Because the TA activation has been proven to occur after the heel-off event and the duration of the TA activation changed with the walking speed (24, 25), a time interval should be implemented after the heel-off event (26). In Thomas et al.’s study, the ankle angle trajectory of the paretic foot was modulated by an iterative learning control method to achieve the desired foot pitch angles (27). The non-linear relationship between the FES settings and the ankle angle influenced the responses of the ankle motion (28). FLC represents a promising technology to handle the non-linearity and uncertainty without the need for a mathematical model of the plant, which has been widely used in robotic control (29). Ibrahim et al. used FLC to regulate the stimulation intensity of the FES (30), and the same control was used on the regulation of the stimulation duration to obtain a maximum knee extension angle in Watanabe et al.’s study (31). However, most closed-loop controls adjust only one stimulation parameter, and few FES controls considered both varying the stimulation intensity and duration while accounting for the changing walking velocities.

In the present study, an intensity- and duration-adaptive FES was established, the FLC and a linear model were used to regulate the stimulation intensity and duration, respectively. The performance of the intensity- and duration-adaptive stimulation (IDAS) was compared with those of stimulation triggered by no stimulation (NS), heel-off stimulation (HOS), and speed-adaptive stimulation (SAS) for poststroke patients walking on a treadmill. The objective of this study is to find an appropriate FES control strategy to realize a more adaptive ankle joint motion for poststroke subjects.

## Materials and Methods

### Subjects

Seven poststroke subjects [five males and two females; mean (±SD) age of the subjects was 46.1 (±11.2) years old; mean (±SD) months after stroke was 9.1 (±7.1) months; Fugl-meyer motor assessment for lower limb was conducted by a physical therapist and the mean value was 25.8 (±3.3) scores] with the ability to walk continuously, and had sufficient passive ankle range of motion to enable their paretic ankle joint to reach at least 5° plantar flexion with the knee flexed at 90° were recruited. Before participating in the experiment, all of the subjects consented to the experimental protocol and provided their written informed consent. The study was approved by the Ethics Committee of the Guangdong Work Injury Rehabilitation Center.

### System Description

**Figure 1:**
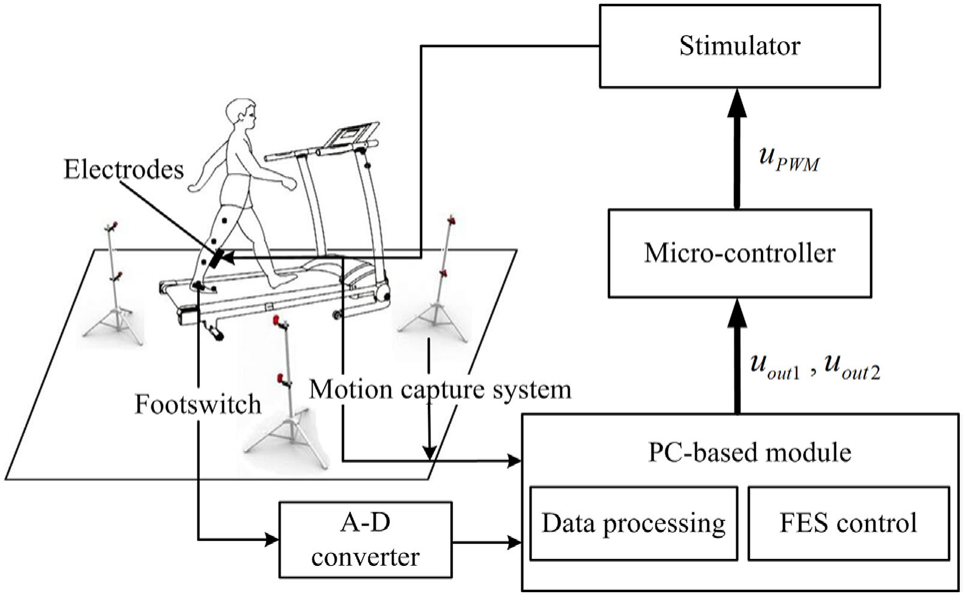
Block diagram of the functional electrical stimulation (FES) system.

The FES rehabilitation system is presented in Figure 1. The system consists of a treadmill (G6425-F3, Beistegui Hermanos, Spain), a functional electrical stimulator (P2-9632, Faisco, China), a micro-controller (STC89C52, Amtel, USA), a motion capture system with four cameras (OptiTrack, NaturalPoint, USA), and a footswitch (B-201, Tekscan, USA). To obtain the positions of each lower limb joint, five markers were attached to them and the three dimensional signals of each marker were captured through the motion capture system. Five markers from top to bottom were placed on the following anatomical reference locations: the mid-thigh sufficiently distal to the hip, the lateral knee joint, the mid-shank sufficiently distal to the knee joint, the lateral malleolus, and the space between the second and third metatarsal heads (32). Additionally, a four segment rigid body model of the lower extremity was implemented to calculate the joint angles (32). A footswitch was placed on the hindfoot to detect the heel-off and heel-strike events, and the signals were recorded on the computer by the A-D converter (see Figure 1). The input signals of the motion capture system and A-D converter were then acquired by the PC-based module, which included the data processing part and the FES control part. The data processing part adopted the input signals to calculate the walking speed, and the joint angle data included the angle signals, the error signals, and the derivative of the error signals. The fuzzy rules and the speed-adaptive rules were adopted to calculate the output signals *u*_out1_ and *u*_out2_, which were the duty ratio and trigger signal, respectively, and then, they were transmitted to the microcontroller. The microcontroller was used to generate the PWM modulation signal, which was delivered to the stimulator to regulate the stimulation intensity and duration. Finally, the stimulator generated the stimulation current to the surface of the TA muscle through two surface electrical stimulation electrodes (M2223, 3M, USA). The pulse amplitude of the stimulator ranged from 0 to 120 mA. The sample rates of the motion capture system and the footswitch were 100 Hz.

### Intensity- and Duration-Adaptive FES

**Figure 2:**
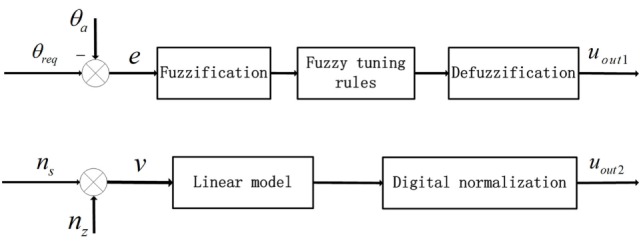
Block diagram of the proposed decentralized modular controller for the control of ankle joint movement. The two inputs were error signals (*e*) and walking speeds (*v*). *n_z_* was the *z* coordinates of the marker on toe and *n_s_* was the average value based on previous five step speeds.

The intensity- and duration-adaptive FES system was implemented during the walking to induce an appropriate ankle joint motion. The structure of the FES system is presented in Figure 2. The output of the FLC and the output of the linear model were transmitted to the micro-controller as two independent signals to determine the intensity and duration of stimulation, respectively. In this study, a change in the stimulation intensity was realized by the modulation of the stimulation pulse amplitude. In the control process, the maximum ankle dorsiflexion angles of each cycle were compared to the desired angle, and the output *u*_out1_ was adjusted on the basis of the error in the next cycle. There are several steps for the proposed FLC system. First, fuzzification was a procedure that translated the inputs into fuzzy linguistic variables. In the proposed FLC, the inputs were the error signal, *e*, and the derivative of the error signal, Δ*e*. Here, *e* was the difference between the actual maximum ankle dorsiflexion angle, θ*_a_*, and the reference value, θ_req_. Additionally, θ_req_ was 4.9°, which was from the normal maximum ankle dorsiflexion angle of human walking (33). The membership degree values of the two inputs can be calculated according to the membership functions, and the functions were defined as triangular membership functions, which were used for the fuzzification process. The input fuzzy sets were acquired by the seven membership functions including negative small (NES), negative medium (NM), negative big (NB), zero (Z), positive small (PS), positive medium (PM), and positive big (PB), and the output fuzzy sets were composed from three membership functions, the PS, PM, and PB (23). Then, we used the predefined fuzzy rule set (Table 1) to elaborate the relationship between the inputs and the outputs of the membership degree value. Finally, the center of the area was implemented as a defuzzification process to transform the membership degree values of the control signal to the actual value of the output *u*_out1_ (34). The *u*_out1_ was transmitted to the microcontroller to control the degree of the PWM wave.

**Table 1:** Fuzzy rules for the adjustment of PWM wave.

*e* Δ*e*	Negative big (NB)	Negative medium (NM)	NES	ZE	Positive small (PS)	Positive medium (PM)	Positive big (PB)
NB	PS	PS	PS	PS	PS	PS	PM
NM	PS	PS	PS	PS	PS	PM	PM
NES	PS	PS	PS	PS	PS	PM	PM
Z	PM	PM	PS	PS	PM	PM	PM
PS	PM	PM	PM	PB	PB	PB	PB
PM	PM	PM	PB	PB	PB	PB	PB
PB	PM	PB	PB	PB	PB	PB	PB

For the linear model, the input was the walking speed, which was calculated by the coordinates of the marker on the toe (35) and the values based on the previous five step speeds were averaged (36). The output of the linear model *u*_out2_ was determined by the time interval of the TA, which was from the heel-off event to the onset timing of the TA activation and the duration of the TA, which was from the onset timing to the terminal timing of the TA activation. In this study, the onset timing and terminal timing were the two trigger signals that the values of *u*_out2_ converted from 0 to 1 and 1 to 0, respectively. They were transmitted to the microcontroller through the serial communication function to control the generation and interrupt of the PWM wave. There were linear relationships between the stimulation duration and walking speed, and between the time interval and walking speed (25). After the correlation analysis and least square curve fitting, the linear models of the walking speed for the time interval and EMG duration were built, and the slope and intercept were −111.7 and 416.9 for the first linear relationship and −213.2 and 877.7 for the second. All of the slopes and intercepts were obtained from healthy subjects in the preliminary experiments.

### Experimental Procedure

During the experiment, the subjects were instructed to hold onto a handrail and walk on a treadmill. Each subject walked on the treadmill at multiple speeds to adapt to the treadmill walking before participating in the experiment. Poststroke subjects were instructed to walk at three speeds: slow, free, and fast. The free speed was the comfortable walking speed on the treadmill, while the fast speed was approximately 25–30% larger than the free walking speed (37). The slow speed was smaller than the free speed with the same proportion. The subjects walked in the four conditions at each speed: no stimulation (NS), FES-assisted walking triggered by the HOS, FES-assisted walking triggered by the SAS, and FES-assisted walking triggered by the IDAS. There were 12 trails for each poststroke subject. The order of the trials was randomly arranged, and a 2-min rest was given between each pair of successive trials to avoid muscle fatigue. In the NS condition, there was no FES during walking. In the HOS condition, the TA stimulation was triggered and terminated by the heel-off and heel-strike events, respectively (38). The stimulation intensity was set as a constant to achieve a neutral ankle angle with the subjects seated (12). In the SAS condition, the duration of the TA stimulation was linear with the walking speed, and the stimulation intensity was the same as the value in the HOS condition. In the IDAS condition, the maximum stimulation intensity was set when reaching the maximum tolerance or achieving the sufficient ankle dorsiflexion angle for each subject (27), and the actual stimulation intensity in the experiment would not exceed this maximum value. The duration of the TA stimulation in this condition was the same as that in the SAS condition. The pulse width and the frequency of stimulation were 390 µs and 40 Hz in the four conditions.

### Data Analysis and Statistical Evaluation

The coordinates of the five markers were acquired to calculate the walking speeds, the maximum ankle dorsiflexion angle, the maximum knee flexion angle during the swing phase, and the ankle plantar flexion angle at the toe-off event. The kinematic signal was filtered by a second-order low-pass Butterworth filter with a cutoff frequency of 15 Hz (39).

The Kolmogorov–Smirnov test was used to assess all of the variables for the normality of the distribution. Then, the one-way analysis of variance with repeated measures (ANOVA) was applied to analyze the influence of the stimulation condition (NS, HOS, SAS, and IDAS) on the ankle dorsiflexion, ankle plantar flexion, and knee flexion angles. If there was a significant difference, then *post hoc* analysis was conducted using the Bonferroni between different conditions. All of the statistical analyses were performed using SPSS 19 (SPSS, Inc., Chicago, IL, USA), and the level of significance was set at 0.05.

## Results

### Automatic Adjustment of Pulse Amplitude

**Figure 3:**
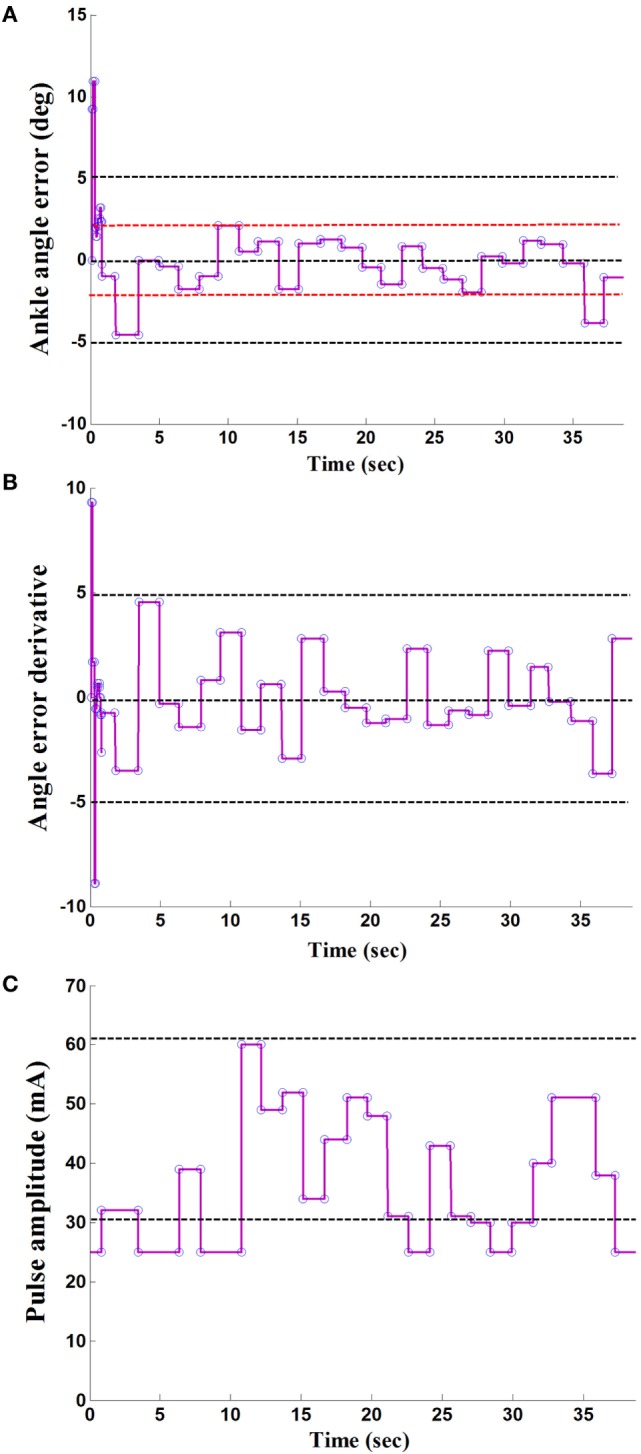
A result of the maximum ankle dorsiflexion angle based on fuzzy logic control: **(A)** the ankle angle error; **(B)** the derivative of angle error; **(C)** the output of stimulation pulse amplitude.

Figures 3A,B showed the error signal between the actual maximum ankle dorsiflexion angle and the reference value, *e*, and the derivative of the error signal, Δ*e*, in the IDAS condition. Figure 3C showed the adaptive pulse amplitude of one poststroke subject during FES-assisted hemiplegic walking at free speed. From Figure 3, there were relatively large errors at the beginning of the walking. After the cycle to cycle adjustment of the pulse amplitude in real-time according to the FLC, the maximum ankle dorsiflexion angles reached the reference value in approximately 5 s and the errors were lower than 2° after the adjustment (The positive ankle angle was equal to the ankle dorsiflexion angle and the negative ankle angle was equal to the ankle plantar flexion angle.) The mean errors between the maximum ankle dorsiflexion angles and reference value were 1.4°, 1.7°, and 1.7° at the slow, free, and fast speed, respectively, in the IDAS condition, which were the smallest values among the four stimulation conditions at each speed (The mean errors were 4.5°, 4.0°, and 4.1° at the slow, free, and fast speed, respectively, and in the NS condition were 2.4°, 2.9°, and 2.8°, at the slow, free, and fast speed, respectively; in the HOS condition, they were 3.0°, 3.6°, and 3.1° at the slow, free, and fast speed, respectively, in the SAS condition).

### Kinematic Data in the Four Stimulation Conditions

**Figure 4:**
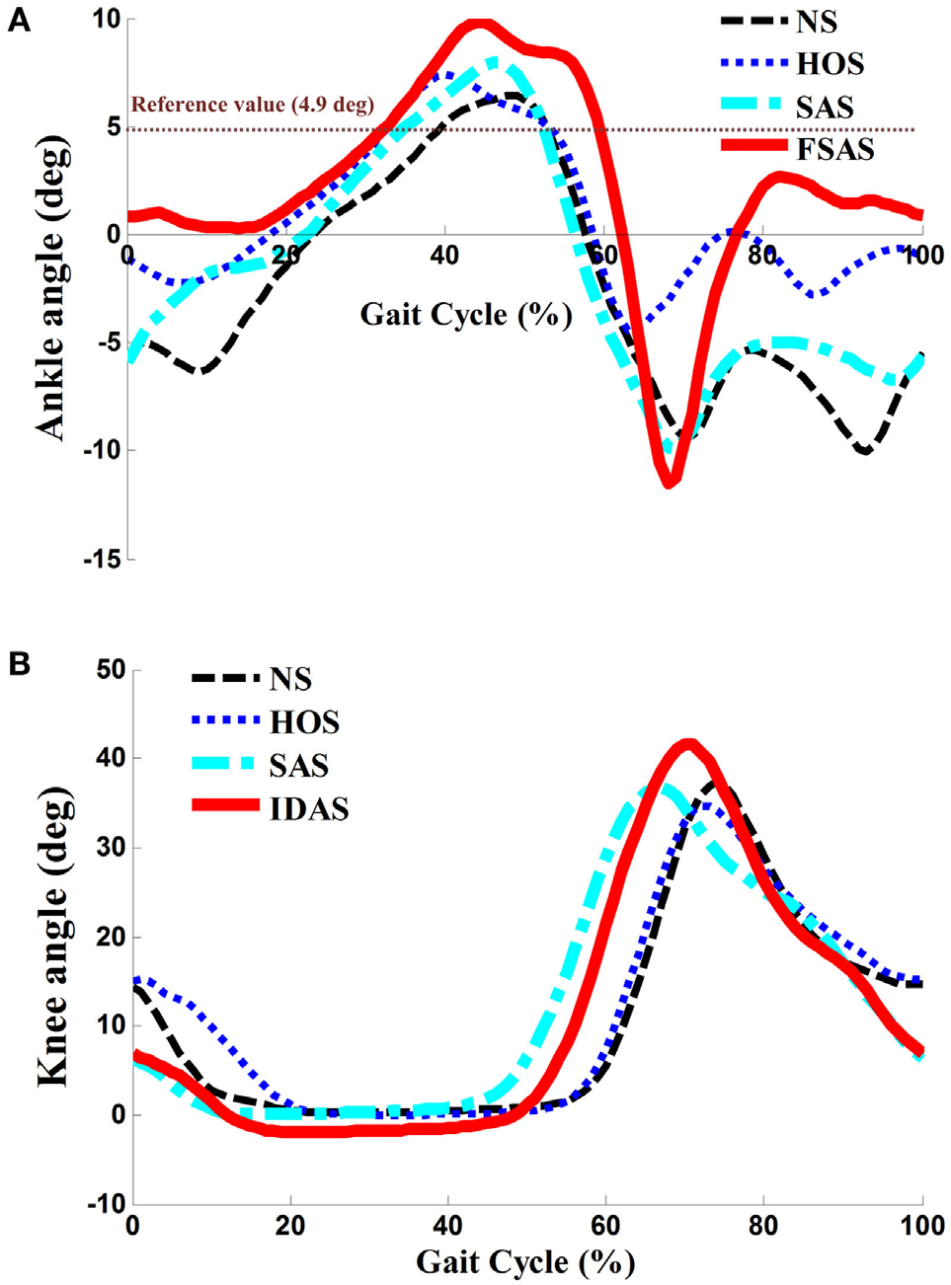
**(A)** Ankle angles during the gait cycle for one poststroke subject at free speed; **(B)** knee angles during the gait cycle for the same poststroke subject at free speed.

**Figure 5:**
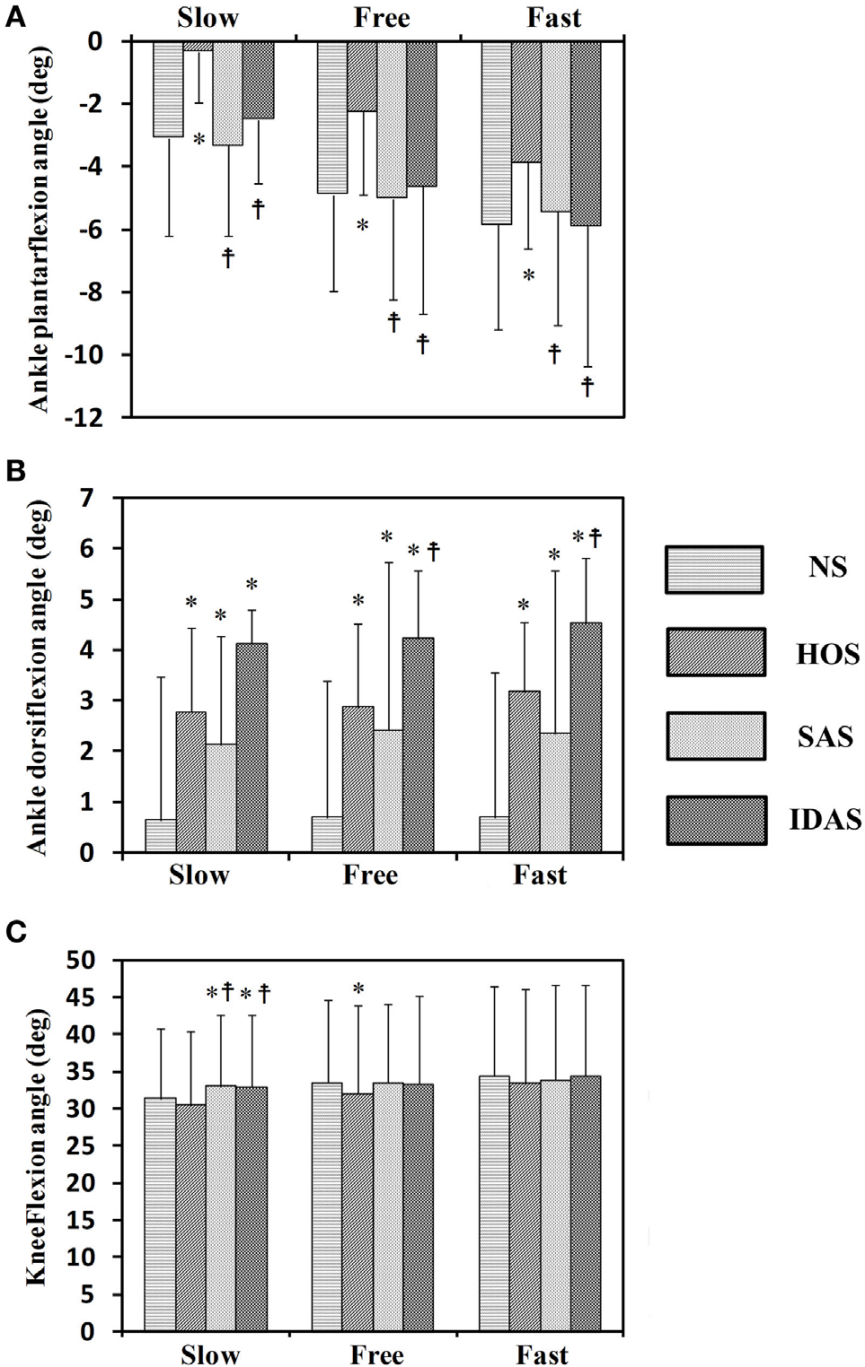
Seven stroke subjects’ results for: **(A)** ankle plantar flexion angles at toe-off event; **(B)** maximum ankle dorsiflexion angles during swing phase; **(C)** maximum knee flexion angles during swing phase; *Significant difference from NS (*P* < 0.05); 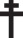
Significant difference from HOS (*P* < 0.05). The error bars represented the SDs.

The Kolmogorov–Smirnov test was applied to the kinematic variables, and the results indicated that all the variables followed a Gaussian distribution (*P* > 0.05). Figure 4 presents the ankle and knee angles of a stroke subject in a gait cycle at free speed. Figure 5 presents the maximum ankle dorsiflexion angles during the swing phase, the ankle plantar flexion angles at the toe-off event, and maximum knee flexion angles of the poststroke subjects during the swing phase in the four walking conditions. Compared to the NS condition, the HOS, SAS, and IDAS conditions all achieved a larger maximum ankle dorsiflexion angle during the swing phase at all of the speeds, and the differences were significant (*P* < 0.05). The maximum ankle dorsiflexion angles in the IDAS condition were closest to 4.9° compared to those in the HOS and SAS conditions at all of the speeds. At the slow, free, and fast speed walking, the average maximum ankle dorsiflexion angles in the IDAS condition were 4.1°, 4.2°, and 4.5°, respectively (Table 2). The values of the maximum ankle dorsiflexion angles in the IDAS condition were larger than those in the HOS and SAS conditions at all of the speeds, and significant differences were found between the IDAS and HOS conditions at the free and fast speeds. The maximum ankle dorsiflexion angles of two subjects were larger than 4.9° at the free speed in the HOS and SAS conditions, which were 5.3° and 6.6°, respectively, and the average maximum ankle dorsiflexion angles were 2.8°, 2.9°, and 3.2° at the slow, free, and fast speed, respectively, in the HOS condition and were 2.1°, 2.4°, and 2.4° at slow, free, and fast speed, respectively, in the SAS condition. The SDs of the maximum ankle dorsiflexion angles in the HOS and SAS conditions were larger than those in the IDAS condition at all of the speeds. The plantar flexion angle in the HOS condition had the minimum value, and the plantar flexion angles in the SAS and IDAS conditions were significantly larger than those in the HOS condition at all of the speeds (Table 3). The plantar flexion angles in the SAS and IDAS conditions were similar to that in the NS condition. The maximum knee flexion angles in the SAS (33.0°) and IDAS conditions (33.0°) were significantly larger than those in the NS (31.4°) and HOS conditions (30.5°) at the slow speed, and there was a significant difference between the NS (33.4°) and HOS conditions (32.1°) at the free speed (Table 4). No significant differences in the maximum knee flexion angle were found in the other two walking conditions (*P* > 0.05).

**Table 2:** The maximum ankle dorsiflexion angles during swing phase.

Ankle dorsiflexion angles (°)		Stimulation conditions			
		
		NS	HOS	Speed-adaptive stimulation	Intensity- and duration-adaptive stimulation
Speed	Slow	0.6	2.8	2.1	4.1
	Free	0.7	2.9	2.4	4.2
	Fast	0.7	3.2	2.4	4.5

**Table 3:** The ankle plantar flexion angles at toe-off event.

Ankle plantar flexion angles (°)		Stimulation conditions			
		
		NS	HOS	Speed-adaptive stimulation	Intensity- and duration-adaptive stimulation
Speed	Slow	−3.1	−0.3	−3.3	−2.5
	Free	−4.9	−2.2	−5.0	−4.6
	Fast	−5.8	−3.9	−5.5	−5.9

**Table 4:** The maximum knee flexion angles during swing phase.

Knee flexion angles (°)		Stimulation conditions			
		
		NS	HOS	Speed-adaptive stimulation	Intensity- and duration-adaptive stimulation
Speed	Slow	31.4	30.5	33.0	33.0
	Free	33.4	32.1	33.5	33.3
	Fast	34.4	33.4	33.9	34.4

## Discussion

Functional electrical stimulation-assisted ankle dorsiflexion was triggered mainly by the fixed stimulation parameters or the modulation of one parameter in previous studies (33, 40, 41). In this study, an intensity- and duration-adaptive FES stimulation was established, and the FLC and linear model were used to regulate the intensity and duration in the IDAS condition, respectively. The performance of the IDAS was compared with those in the NS, HOS, and SAS conditions.

Although higher maximum ankle dorsiflexion angles in the HOS and SAS conditions than that in the NS condition were found, the stimulation intensity was set to be a constant in these two conditions, which could not account for the changing ankle angles during the gait cycles. If the intensity was too strong, then an exaggerated foot lift would be caused during walking. Overlarge maximum ankle dorsiflexion angles of two subjects were observed in the HOS and SAS conditions, which might cause the muscle to fatigue rapidly and the subjects to feel discomfort (14). If the intensity was too weak, then, it might not yield a sufficiently large maximum ankle dorsiflexion angle during walking. The insufficient maximum ankle dorsiflexion angles would lead to decreased foot clearance, which results in an increasing risk of falls (42, 43). Delivering FES in the IDAS condition was intensity adaptive, which can account for changing the ankle angles during the gait cycles. As demonstrated by the results in Figures 4 and 5, the SDs of the maximum ankle dorsiflexion angles in the IDAS condition were smallest compared to those in other conditions at all of the speeds, and they were closer to the data of the healthy subjects described in the previous research (44), which would produce a more safe foot lift during walking. The larger variance in other conditions might be caused by the loss of muscle control ability during walking due to the fixed stimulation intensity (36). Although the ankle plantar flexion angle was not worsened in the SAS condition, a smaller maximum ankle dorsiflexion angle was observed in the SAS condition than that in the HOS condition. This difference would cause a smaller ankle excursion influencing the stability of walking, and a similar result was also shown in Kesar et al.’s study (45). Because the TA muscles were activated with the same stimulation intensity in these two conditions, greater stimulation intensity for the TA muscle would be required to generate a larger maximum ankle dorsiflexion angle during the swing phase in the SAS condition. At the same time, the stimulation intensity should be regulated to an appropriate magnitude but not an excessively high level, to avoid muscle fatigue.

In addition to the stimulation intensity being important, the stimulation duration was also essential for the FES parameterization. Liberson et al. and Springer et al. started and terminated the stimulation using heel-off events and heel-strike events, respectively (46, 47). Although there was improvement in the maximum ankle dorsiflexion angle in the HOS condition, the ankle plantar flexion angle was worsened at the toe-off event in the HOS condition when compared to those in other conditions, which suggest that they were not the optimal timing for the stimulation (shown in Figure 5). On the other aspect, the FES triggered by the HOS condition led to the decreased maximum knee flexion angles at the swing phase when compared to those in the other conditions, and similar results were also discovered in Bhadra et al.’s study (48). The decreased maximum knee flexion angles were related to the decreased ankle plantar flexion angles at the toe-off event. Additionally, the decreased ankle plantar flexion angle would result in a decreased forward propulsive force (12). There were improvements in the maximum ankle dorsiflexion angles, ankle plantar flexion angles, and maximum knee flexion angles at all of the speeds in the IDAS condition, which suggested that the proposed control was more suitable for the poststroke subjects’ rehabilitation.

Although both the maximum ankle dorsiflexion and ankle plantar flexion angle have improved in the IDAS condition, there were still some limitations. Only movement in the sagittal plane was considered in this study. Further studies will be conducted to address the undesired foot eversion/inversion in the frontal plane for poststroke subjects. The maximum ankle dorsiflexion angle, ankle plantar flexion angle, and maximum knee flexion angle were applied to evaluate the performance of FES-assisted walking, and more evaluation methods will be investigated in a future study, such as the differences in the pattern of ankle/knee motion between the poststroke subjects and healthy subjects. Peri et al. combined cycling with FES to enhance functional improvements for poststroke subjects (49). In our future study, the combination of the proposed FES control strategy and cycling will be investigated to further confirm its clinical effectiveness.

## Conclusion

The purpose of this study was to apply an intensity- and duration-adaptive FES control to determine the output of a stimulator and to explore whether the proposed control strategy was appropriate or not for poststroke subjects’ rehabilitation. The results showed that there were improvements in both the maximum ankle dorsiflexion angle and ankle plantar flexion angle in the IDAS condition compared to those in the other conditions. In the future, more studies on electrode placements are needed to validate whether the ankle joint angle is influenced by the precision of the electrode placements. Investigations could also be conducted with more poststroke subjects in a clinical experiment to validate the effectiveness in rehabilitation training.

## Ethics Statement

Before participating in the experiment, all of the subjects consented to the experimental protocol and provided their written informed consent before participating in the study, and the study was approved by the Ethics Committee of the Guangdong Work Injury Rehabilitation Center.

## Author Contributions

GC and RS designed the study, and GC conducted the experiments. ZS, YZ, and XW helped to perform the experiments. GC and RS analyzed the data, interpreted the results, and drafted and revised the manuscript. All of the authors approved the final version of the manuscript.

## Conflict of Interest Statement

The authors declare that the research was conducted in the absence of any commercial or financial relationships that could be construed as a potential conflict of interest.

## Abbreviations

FES, functional electrical stimulation; TA, tibialis anterior; NS, no stimulation; HOS, heel-off stimulation; SAS, speed-adaptive stimulation; IDAS, intensity- and duration-adaptive stimulation; FLC, fuzzy logic control.

## References

[1] Lloyd-Jones D Adams R Carnethon M Simone D Ferguson TB Flegal K A report from the American Heart Association statistics committee and stroke statistics subcommittee. Circulation (2009) 119(3):480–6. 10.1161/CIRCULATIONAHA.108.191259 19171871

[2] Arene N Hidler J. Understanding motor impairment in the paretic lower limb after a stroke: a review of the literature. Top Stroke Rehabil (2009) 16(5):346–56. 10.1310/tsr1605-346 19903653

[3] Neckel N Pelliccio M Nichols D Hidler J. Quantification of functional weakness and abnormal synergy patterns in the lower limb of individuals with chronic stroke. J Neuroeng Rehabil (2006) 3(1):17. 10.1186/1743-0003-3-17 16857059 PMC1553458

[4] Granger CV Hamilton BB Gresham GE The stroke rehabilitation outcome study – part I: general description. Arch Phys Med Rehabil (1988) 69(7):506–9. 3389991

[5] Simons CD van Asseldonk EH Van der KH Geurts AC Buurke JH. Ankle-foot orthoses in stroke: effects on functional balance, weight-bearing asymmetry and the contribution of each lower limb to balance control. Clin Biomech (2009) 24(9):769–75. 10.1016/j.clinbiomech.2009.07.006 19665825

[6] Van Peppen RP Kwakkel G Wooddauphinee S Hendriks HJ Pj VDW Dekker J. The impact of physical therapy on functional outcomes after stroke: what’s the evidence? Clin Rehabil (2004) 18(8):833–62. 10.1191/0269215504cr843oa 15609840

[7] Saleh S Fluet G Qiu Q Merians A Adamovich SV Tunik E. Neural patterns of reorganization after intensive robot-assisted virtual reality therapy and repetitive task practice in patients with chronic stroke. Front Neurol (2017) 8:452. 10.3389/fneur.2017.00452 28928708 PMC5591400

[8] Ring H Treger I Gruendlinger L Hausdorff JM. Neuroprosthesis for footdrop compared with an ankle-foot orthosis: effects on postural control during walking. J Stroke Cerebrovasc Dis (2009) 18(1):41–7. 10.1016/j.jstrokecerebrovasdis.2008.08.006 19110144

[9] Pilkar R Ramanujam A Nolan KJ. Alterations in spectral attributes of surface electromyograms after utilization of a foot drop stimulator during post-stroke gait. Front Neurol (2017) 8:449. 10.3389/fneur.2017.00449 28900414 PMC5581808

[10] Lyons GM Sinkjaer T Burridge JH Wilcox DJ. A review of portable FES-based neural orthoses for the correction of drop foot. IEEE Trans Neural Syst Rehabil Eng (2002) 10(4):260–79. 10.1109/TNSRE.2002.806832 12611364

[11] Tm K As W Sa BM Novel FES system to stimulate both dorsi- and plantar-flexor muscles during stroke gait. 12th Annual Conference of the International FES Society Philadelphia, PA (2007).

[12] Kesar TM Perumal R Jancosko A Reisman DS Rudolph KS Higginson JS Novel patterns of functional electrical stimulation have an immediate effect on dorsiflexor muscle function during gait for people poststroke. Phys Ther (2010) 90(1):55–66. 10.2522/ptj.20090140 19926681 PMC2802826

[13] Riener R Fuhr T Schneider J On the complexity of biomechanical models used for neuroprostheses development. J Mech Med Biol (2002) 2(03n04):389–404. 10.1142/S0219519402000459

[14] Lynch CL Popovic MR Functional electrical stimulation. IEEE Control Syst (2008) 28(2):40–50. 10.1109/MCS.2007.914689

[15] Spaich EG Bøg MF Erkocevic E Smidstrup A Andersen OK Nielsen JF Gait orthosis lokomat combined with functional electrical stimulation for foot drop correction: a feasibility study. Springer (2014) 7:751–7. 10.1007/978-3-319-08072-7_104

[16] Lee YH Yong SY Kim SH Kim JH Shinn JM Kim Y Functional electrical stimulation to ankle dorsiflexor and plantarflexor using single foot switch in patients with hemiplegia from hemorrhagic stroke. Ann Rehabil Med (2014) 38(3):310–6. 10.5535/arm.2014.38.3.310 25024953 PMC4092170

[17] Melo PL Silva MT Martins JM Newman DJ. Technical developments of functional electrical stimulation to correct drop foot: sensing, actuation and control strategies. Clin Biomech (2015) 30(2):101–13. 10.1016/j.clinbiomech.2014.11.007 25592486

[18] Rouhani H Same M Masani K Li YQ Popovic MR. PID controller design for FES applied to ankle muscles in neuroprosthesis for standing balance. Front Neurosci (2017) 11:347. 10.3389/fnins.2017.00347 28676739 PMC5476782

[19] Negård N-O Schauer T Kauert R Raisch J An FES-assisted gait training system for hemiplegic stroke patients based on inertial sensors. IFAC Proc Vol (2006) 39(18):315–20. 10.3182/20060920-3-FR-2912.00058

[20] Benedict GA Ruiz VF, editors. A portable gait analysis and correction system using a simple event detection method. IEEE International Conference on Systems, Man and Cybernetics Yasmine Hammamet, Tunisia (2002).

[21] Cho W Sabathiel N Ortner R Lechner A Irimia DC Allison BZ Paired associative stimulation using brain-computer interfaces for stroke rehabilitation: a pilot study. Eur J Transl Myol (2016) 26(3):219–22. 10.4081/ejtm.2016.6132 27990240 PMC5128973

[22] Laursen CB Nielsen JF Andersen OK Spaich EG. Feasibility of using Lokomat combined with functional electrical stimulation for the rehabilitation of foot drop. Eur J Transl Myol (2016) 26(3):268–73. 10.4081/ejtm.2016.6221 27990246 PMC5128979

[23] Nekoukar V Erfanian A. A decentralized modular control framework for robust control of FES-activated walker-assisted paraplegic walking using terminal sliding mode and fuzzy logic control. IEEE Trans Biomed Eng (2012) 59(10):2818–27. 10.1109/TBME.2012.2208963 22868526

[24] Monaghan CC van Riel WJ Veltink PH. Control of triceps surae stimulation based on shank orientation using a uniaxial gyroscope during gait. Med Biol Eng Comput (2009) 47(11):1181–8. 10.1007/s11517-009-0539-8 19830470 PMC2768796

[25] Byrne CA O’Keeffe DT Donnelly AE Lyons GM. Effect of walking speed changes on tibialis anterior EMG during healthy gait for FES envelope design in drop foot correction. J Electromyogr Kinesiol (2007) 17(5):605–16. 10.1016/j.jelekin.2006.07.008 16990012

[26] Burridge JH Taylor PN Hagan SA Wood DE Swain ID. The effects of common peroneal stimulation on the effort and speed of walking: a randomized controlled trial with chronic hemiplegic patients. Clin Rehabil (1997) 11(3):201–10. 10.1177/026921559701100303 9360032

[27] Seel T Werner C Raisch J Schauer T Iterative learning control of a drop foot neuroprosthesis – generating physiological foot motion in paretic gait by automatic feedback control. Control Eng Pract (2016) 48:87–97. 10.1016/j.conengprac.2015.11.007

[28] Miura N Watanabe T Sugimoto S Seki K Kanai H. Fuzzy FES controller using cycle-to-cycle control for repetitive movement training in motor rehabilitation. Experimental tests with wireless system. J Med Eng Technol (2011) 35(6–7):314–21. 10.3109/03091902.2011.591480 21767134

[29] Teodorescu HNN Kandel A Jain LC Fuzzy and Neuro-Fuzzy Systems in Medicine. Boca Raton, FL: CRC Press, Inc. (1998).

[30] Ibrahim BSKK Tokhi MO Huq MS Gharooni SC, editors. Fuzzy logic based cycle-to-cycle control of FES-induced swinging motion. International Conference on Electrical, Control and Computer Engineering Pahang, Malaysia (2011). p. 60–4.

[31] Watanabe T Masuko T Arifin A Yoshizawa M A feasibility study of fuzzy FES controller based on cycle-to-cycle control: an experimental test of knee extension control. IEICE Trans Inf Syst (2008) 91(3):865–8. 10.1093/ietisy/e91-d.3.865

[32] Kadaba MP Ramakrishnan HK Wootten ME Measurement of lower extremity kinematics during level walking. J Orthop Res (1990) 8(3):383–92. 10.1002/jor.1100080310 2324857

[33] Arifin A Watanabe T Hoshimiya N Fuzzy controller for cycle-to-cycle control of swing phase of FES-induced hemiplegic gait: a computer simulation in two-joints control. Int Conf IEEE Eng Med Biol Soc (2003) 2:1519–22. 10.1109/IEMBS.2003.1279636

[34] Yang J Su H Li Z Ao D Song R Adaptive control with a fuzzy tuner for cable-based rehabilitation robot. Int J Control Automation Syst (2016) 14(3):865–75. 10.1007/s12555-015-0049-4

[35] Zeni JA Jr Richards JG Higginson JS. Two simple methods for determining gait events during treadmill and overground walking using kinematic data. Gait Posture (2008) 27(4):710–4. 10.1016/j.gaitpost.2007.07.007 17723303 PMC2384115

[36] Chen M Wu B Lou X Zhao T Li J Xu Z A self-adaptive foot-drop corrector using functional electrical stimulation (FES) modulated by tibialis anterior electromyography (EMG) dataset. Med Eng Phys (2013) 35(2):195–204. 10.1016/j.medengphy.2012.04.016 22621781

[37] Tyrell CM Roos MA Rudolph KS Reisman DS. Influence of systematic increases in treadmill walking speed on gait kinematics after stroke. Phys Ther (2011) 91(3):392–403. 10.2522/ptj.20090425 21252308 PMC3048817

[38] Pappas IPI Keller T Mangold S Popovic MR Dietz V Morari M A reliable gyroscope-based gait-phase detection sensor embedded in a shoe insole. IEEE Sens J (2004) 4(2):268–74. 10.1109/JSEN.2004.823671

[39] Van den Bogert A De Koning J On optimal filtering for inverse dynamics analysis. Proceedings of the IXth Biennial Conference of the Canadian Society for Biomechanics Vancouver (1996). p. 214–5.

[40] Heller BW Clarke AJ Good TR Healey TJ Nair S Pratt EJ Automated setup of functional electrical stimulation for drop foot using a novel 64 channel prototype stimulator and electrode array: results from a gait-lab based study. Med Eng Phys (2013) 35(1):74–81. 10.1016/j.medengphy.2012.03.012 22559959

[41] Mourselas N Granat MH Correction of drop foot using a fuzzy logic controlled miniature stimulator. Proc. 5th Ann. IFESS Conf Scotland (2000).

[42] Sabut SK Kumar R Mahadevappa M, editors. Design of an insole embedded foot pressure sensor controlled FES system for foot drop in stroke patients. International Conference on Systems in Medicine and Biology Kharagpur, India (2010). p. 237–41.

[43] Lundinolsson L Nyberg L Gustafson Y “Stop walking when talking” as a predictor of fall in elderly people. Lancet (1997) 349(9052):617 10.1016/S0140-6736(97)24009-2 9057736

[44] Chen M Wang QB Lou XX Xu K Zheng XX, editors. A foot drop correcting FES envelope design method using tibialis anterior EMG during healthy gait with a new walking speed control strategy. Engineering in Medicine & Biology Society. Buenos Aires, Argentina (2010). p. 4906–9. 10.1109/IEMBS.2010.5627250 21096659

[45] Kesar TM Perumal R Reisman DS Jancosko A Rudolph KS Higginson JS Functional electrical stimulation of ankle plantar- and dorsi-flexor muscles: effects on post-stroke gait. Stroke (2009) 40(12):3821–7. 10.1161/STROKEAHA.109.560375 19834018 PMC2827197

[46] Springer S Vatine JJ Lipson R Wolf A Laufer Y Effects of dual-channel functional electrical stimulation on gait performance in patients with hemiparesis. Sci World J (2012) 2012(2):530906–13. 10.1100/2012/530906 PMC3477556 23097635

[47] Liberson WT Holmquest HJ Scot D Dow M Functional electrotherapy: stimulation of the peroneal nerve synchronized with the swing phase of the gait of hemiplegic patients. Arch Phys Med Rehabil (1961) 42(8):101–5. 13761879

[48] Bhadra N Kilgore KL Peckham PH. Implanted stimulators for restoration of function in spinal cord injury. Med Eng Phys (2001) 23(1):19–28. 10.1016/S1350-4533(01)00012-1 11344004

[49] Peri E Ambrosini E Pedrocchi A Ferrigno G Nava C Longoni V Can FES-augmented active cycling training improve locomotion in post-acute elderly stroke patients? Eur J Transl Myol (2016) 26(3):187–92. 10.4081/ejtm.2016.6063 27990234 PMC5128967

